# Hernia

**Published:** 1890-03-15

**Authors:** 


### HERNIA.

Dr. Joseph D. Bryant, of New York, gives in a recent *New York Medical Record*, an article on the treatment of Hernia. Dr. Bryant traced the history of the treatment of hernia from the earliest times, mentioning that Fabricius Hildanus in the sixteenth century was the first to discard the rude wood and iron supports previously used by the invention of a flexible steel spring. Dr. Bryant remarks that the greater number of medical men of the present day are blissfully ignorant of the truss armamentarium now in the market, also of the measurement, means, and method of fitting them properly to patients (an observation which we do not think applicable to British practitioners, although the next observation is more pertinent). "The patients thus afflicted are relegated to instrument makers." That ruptures are sometimes cured by the proper use of trusses there is no doubt, but the number of substantial cures resulting from trusses is small. Dr. Bryant advises the use of three destinctive trusses, one for day use, another impervious to water for bathing, and another for the hours of rest. The establishment of the habit of assuming the recumbent position while making the interchange is the only safe method of procedure. Dr. Bryant might also have added that if a truss chafes at first, the skin should be bathed with alum water; that all trusses should be washable; that it is generally desirable for a person, although only ruptured on one side, to wear a double truss, as there is often a corresponding weakness on the unruptured side, and with a truss upon one groin a greater strain is thrown upon the other; that unless a truss fulfils all requirements it may do injury.

Dr. Bryant next proposes the question, which is the best method for the radical cure, the "open or the unopen?" The most substantial obstacle to the escape of hernia exists at the internal ring. And the chief object consists in strengthening the internal ring and contiguous parts. The method of MacEwan is compared with the open plan, the latter method is advocated by the author. In this method the neck of the sac is closed by a ligature. The sac is then cut off, the aponeurosis of the external oblique slit up to opposite the internal ring, and the entire wound caused to heal by granulation. This the author regards as the most rational and simplest method for the radical cure of hernia. The author further thinks that the radical cure should be practised as an addendum to herniotomy. Now many operations have been devised for the radical cure of hernia. In our opinion the plan known as Wood's method to be found described in most of our surgical works, is the operation generally applicable and successful.

